# Pediatric Emergency Department Management in Acute Poisoning—A 2-Year Retrospective Study

**DOI:** 10.3390/jpm13010106

**Published:** 2023-01-03

**Authors:** Mihaela Corlade-Andrei, Paul Lucian Nedelea, Theodora Daniela Ionescu, Tamara Solange Rosu, Alexandra Hauta, Gabriela Raluca Grigorasi, Teofil Blaga, Ivona Sova, Ovidiu Tudor Popa, Diana Cimpoesu

**Affiliations:** 1 Faculty of Medicine, University of Medicine and Pharmacy “Gr. T. Popa”, 700115 Iași, Romania; 2 St Spiridon Emergency University Hospital, 700111 Iași, Romania; 3 St Mary Emergency Hospital for Children, 700309 Iași, Romania

**Keywords:** children, pediatric, poisoning, toxics, emergency care

## Abstract

(1) Background: Poisonings in children are common reasons for addressing ED and can potentially have serious complications. Our research aims to review risk factors leading to poisoning in children. (2) Methods: A retrospective review of all pediatric poisoning cases addressing the Children’s emergency department of St Mary Hospital over a two-year period was performed. (3) Results: We collected data on 797 children admitted for acute poisoning. The highest incidence identified was in the 12–18 and 1–3-year-old age groups. The distribution of voluntary versus unintentional poisonings was relatively balanced: 50.19% versus 47.43% (for some cases the type of intoxication remained unknown). Exposure to the toxic substance by ingestion was significant compared to the other routes, with an incidence of 87.1%. Acute poisoning happened at home in 70.4% of cases. A known risk factor before reaching the ED was present in 13.04%. (4) Conclusions: Our study showed a greater risk for acute poisoning in children between 1–3 years of age, and adolescents over 12 years. Identifying and documenting epidemiological aspects and other variables is important for establishing preventive measures and for therapeutic conduct. Adequate risk stratification and preventive measures involving closer supervision of minors or cognitive-behavioral programs can prevent voluntary intoxication.

## 1. Introduction

Acute poisoning is a major global health concern, as it is one of the leading causes of morbidity and infant mortality worldwide. Children are particularly vulnerable to acute poisoning, as they are more likely to explore their environment by putting things in their mouths and have less-developed immune systems. Given the current social and economic context, pediatric patients’ exposure to toxic substances is constantly changing, both in terms of the types of toxins involved and the context of scientific research.

Despite significant progress in identifying toxicants, clinical skills in recognizing toxidromes by combining data from the patient’s history (if available), physical examination, and basic investigations are still essential for the management of children with suspected intoxication in the emergency department (ED). Toxidromes refer to the characteristic signs and symptoms that are associated with the ingestion of specific types of toxins. By recognizing the toxidrome, healthcare providers can better identify the toxicant and provide appropriate treatment.

Intoxication in children, whether or not it results in multisystem damage, presents special diagnostic and treatment challenges, regardless of the route of exposure. Children may be exposed to toxic substances through ingestion, inhalation, injection, or absorption through the skin. Each route of exposure can have different signs and symptoms, and different treatment approaches may be needed.

Our research aims to identify the risk factors that lead to intoxication in children, as well as the main types of toxic substances involved. By understanding these factors, we can better prevent and manage acute poisoning in children. In addition to analyzing these factors, we also aim to provide recommendations for preventive measures specific to our area.

## 2. Materials and Methods

The retrospective study included patients under the age of 18, with a history of exposure to toxic substances, who benefited from medical care in the ED of the St Mary Emergency Clinical Hospital for Children, Iași. The data collection was carried out over a period of 24 months (1 January 2019 to 31 December 2020) by analyzing the Intoxication Register and observation sheets, as well as the data from the existing InfoWord system in the medical unit. Patients with incomplete data and patients with local or systemic allergic reactions after insect/snake bites were excluded from the study. Statistical analysis of the data was performed using IMB SPSS STATISTICAL version 26, with the identification of variables defined quantitatively and qualitatively, depending on the case. The *p* value below 0.05 was considered statistically significant.

## 3. Results

During the analyzed period, in the ED of the St Mary Emergency Clinical Hospital for Children, Iași, 797 pediatric patients presented with intoxications, representing 1.43% of the total cases (797 out of 55,599).

Analyzing the seasonal aspect—the seasonal distribution of intoxications—we found for 2019 a symmetrical distribution on the spring seasons (27.96%), summer (25.95%), autumn (26.85%) and a slight decrease in winter (19.24%). In 2020, the distribution showed a predominance of cases in the winter season (29.14%), with similarity in the summer season (25.71%) and autumn (24.29%), and a lower incidence in the spring of that year (20.86%).

The analysis related to the age parameter showed the highest incidence of intoxications in the age group 12–18 years (49.44%, 394 cases), followed by the age group 1–3 years (23.34%, 186 cases), as shown in Table 1.

Gender does not reveal statistically significant differences in the distribution of intoxication in the studied group (51.7% male and 48.3% female), but it does highlight differences by age group. At a young age (1–7 years), males predominate (167 out of 293 cases), motivated by their increased curiosity and the need to explore the environment in which they live, while in patients in the 12–18-year-old age group, females predominate (213 out of 394 cases). For patients in the 7–12-year-old age group, the distribution is relatively uniform across the two genders.

With regard to the environment of origin, most patients (65.9%) come from rural areas (525 out of 797 patients). The analysis of the correlations between demographic factors (age, gender and residence) showed there is a significant correlation between the patient’s age and gender, and between the patient’s gender and residence, the significance threshold being below 0.001 as showed in Table 2.

The correlation coefficient r between sex and age has a negative value of −0.124, indicating the significance of the correlation, which is inversely proportional. At a young age, males predominate and, as age increases, females predominate (significance threshold below 0.01 in 797 subjects). The correlation coefficient r between environment and sex has a positive value of 0.104, indicating the significance of the correlation, which is directly proportional. The rural environment is representative for both sexes (significance threshold below 0.01 in 797 subjects). There is no significant correlation between age and environment.

**Figure 1:**
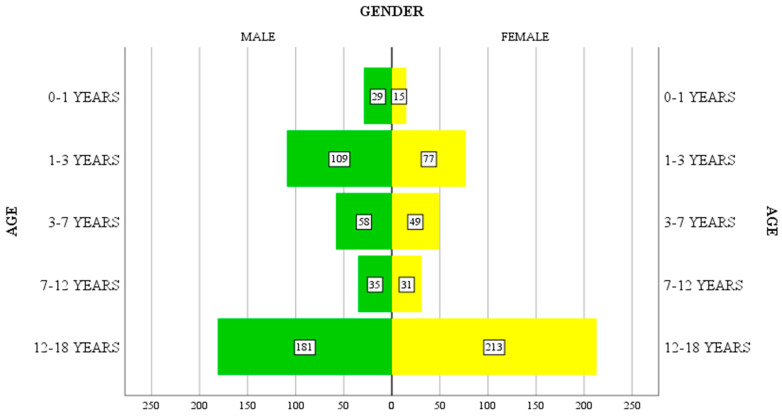
The correlation between the age groups and the sex of the patients.

Although the gender distribution of patients is similar (412 boys and 385 girls), the correlation between patients’ gender and age group (as showed in Figure 1) indicates a slightly higher incidence of female intoxication for the 12–18-year-old group (213 girls compared to 181 boys), consisting of 54% of patients in this age group.

The rural environment is prevalent for all age groups, consisting of 525 patients out of 797 total (65.8%). The higher incidence of cases in rural areas is also reflected in the distribution for both sexes, such that 70% of boys (291 out of a total of 412 boys) and 60.7% of girls (234 out of a total of 385 girls) come from rural areas.

From the analysis of the observation sheets, with the limits determined by the possibility that the cases were not documented, 104 (13.04%) particular situations were identified as socially “stigmatized” cases. There is a possibility that the number of such cases may be higher, but these have not been documented in the observation sheets. There were 24 patients notified as school drop-outs, with 11 designated as social cases (disorganized families, family alcoholism, extremely poor environmental and living conditions) and 8 patients coming from care centers (institutionalized). All were in the 12–18 age group, presenting voluntary intoxication with substances including alcohol, psychodysleptic substances, marijuana, opiates and other drugs. There were also 24 patients who presented with emotional and conduct disorders, and 10 patients with depressive-anxiolytic syndrome, all cases being diagnosed prior to presentation. In 20 cases, the patients had liminal intellect up to severe mental retardation, while 3 patients with autism were victims of accidental intoxication (2 cases in the age group 3–7 years and 1 case in the age group 7–12 years), and 1 patient with Down syndrome and 1 patient with polymalformative syndrome and mental retardation presented with iatrogenic intoxication. Out of a total of 385 female patients, 3 patients (0.779%) were pregnant in the first trimester of pregnancy (2 cases in rural areas and one case in an urban area), all three with voluntary intoxication for suicide reasons (toxicants involved included anticonvulsants, nonsteroidal anti-inflammatory drugs, and hydrocarbons).

**Figure 2:**
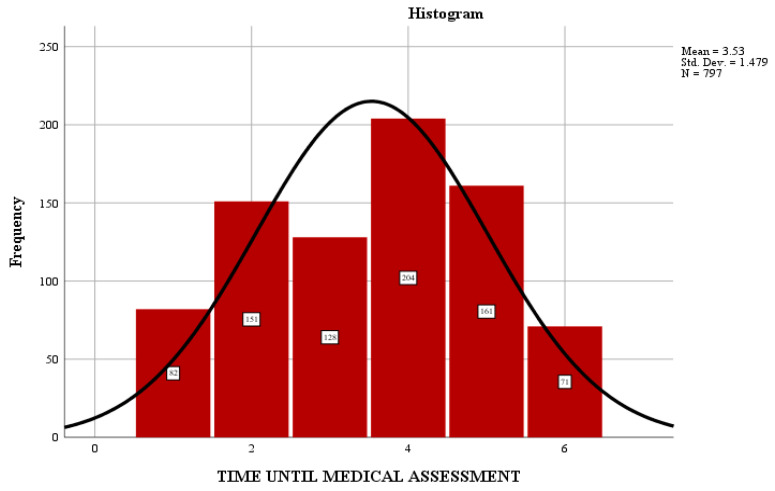
Distribution of cases according to the time interval from exposure to ED arrival.

The time elapsed from the time of exposure to the toxicant until the time of addressing the ED directly influences the provision of specific medical care and consequently the patient’s response, hospitalization period and severity of the case. Most patients went to the ED a long time after exposure to the toxic substance, with an average of 3.53 h as shown in Figure 2.

Some 25.6% of patients were evaluated in the first 4 h after exposure, and 20.3% after 4 h. A small number of patients showed up in the first hour after exposure (10.29% of cases). As a significant percentage of the study group is from rural areas, accessibility to specialist medical services is delayed due to distance, means of transport, and educational status.

A higher percentage of intoxications (70.4%) took place at the patient’s home (561 cases out of a total of 797), most of them being accidental intoxications. In situations where the event occurred at home, 14% of patients arrived at the hospital in the first hour, unlike the poisonings that occurred in other locations. In such situations, the percentage was 1.2%. Presentation in the medical unit in the first 4 h after the exposure was 23.1% for the situations in which exposure to the toxicant took place at home, compared to 31.8% for other locations. For those presenting after 4 h, 13.9% of patients had been exposed at home, compared to 35.3% who had exposure elsewhere. There is no definite correlation between the time elapsed until presentation in ED and the type of intoxication, regardless of the location of the event.

Regarding the circumstances of exposure to the toxic substance, these were classified as accidental, voluntary, iatrogenic or remained unknown (unconscious patient or conscious patient but refusing to provide information). Iatrogenic intoxications, although a small percentage overall (18 cases, 2.26%), should not be ignored, as this circumstance raises both medical practice problems and self-medication problems. The toxins in this type of intoxication included oral and injectable antiemetics (metoclopramide), oral and inhaled bronchodilators (salbutamol), iron supplements, paracetamol, and other nonsteroidal anti-inflammatory drugs, antibiotics, antipsychotics (haloperidol), etc. The age group most exposed to iatrogenic intoxication was 7–12 years. The reults of the type of poisoning in the study group are shown in Table 3.

The routes of exposure identified in the patients in the study group included the following: by ingestion, by inhalation, by direct skin contact, by injection, by multiple routes, or the toxicant could not be identified in the first stage and as a result, neither could the route of exposure.

Analyzing the type of intoxication versus the route of exposure, it is found that ingestion is the most common route, with a similar distribution on all categories of intoxication—87.5% of voluntary intoxications, 87% of accidental intoxications, and 83.33% of iatrogenic cases. Exposure by inhalation was identified in lower percentages, both for voluntary and accidental intoxications (11.38% and 12.75%, respectively).

**Figure 3:**
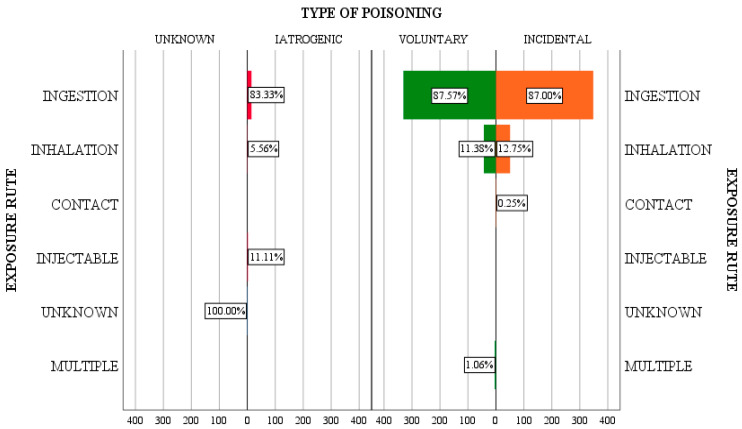
The incidence of poisoning in correlation to the exposure route.

Exposure to the toxic substance by ingestion was significant compared to the other routes, with an incidence of 87.1% (694 cases out of a total of 797), followed by inhalation in 11.9% (95 patients out of a total of 797). In 4 cases, the patients presented multiple routes of exposure, namely ingestion and inhalation, with toxic ethyl alcohol and psychodysleptic substances. At the level of the studied group, the oral route of administration predominated in the 12–18 age group (47.98%), followed by those aged 1–3 years (25.5%). The two sexes had a similar distribution. The results of the incidence of poisoning correlated to the manner of exposure are shown in Figure 3.

**Figure 4:**
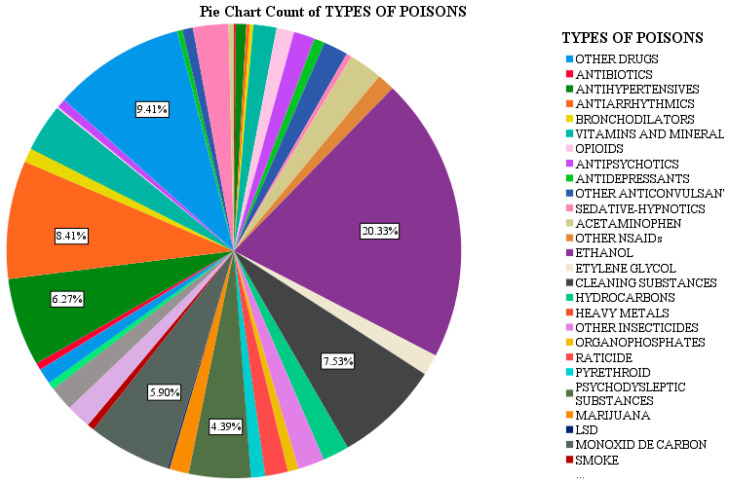
Types of poisons in the studied batch.

Following the analysis of the group of 797 patients, it was found there is a diversity of toxins to which children are exposed, with a higher incidence for certain types of toxins, as shown in Figure 4.

The most common toxicant found in the study group (21.4% of cases) was ethyl alcohol, alone or in combination with other toxics (antipsychotics, anticonvulsants, drugs). The highest incidence belonged to the 12–18 age group (94.7%). In the 1–3-year-old age group (1.2%), patients came from disadvantaged backgrounds, with alcoholic families, while in those aged 3–7 years (1.7%), the ingestion was accidental due to the storage of ethyl alcohol in inappropriate containers (e.g., juice bottles). Prevalence in males predominates this type of intoxication, and the dominant environment is the rural one (69%, 118 cases out of 171, versus 31% for the urban environment).

The drugs consumed by the patients in the study group were psychodysleptic substances, marijuana and LSD in 47 cases (5.89% of all poisonings), most residing in urban areas (57.4%), male (72.3%), and 12–18 years of age (97.9%). One case was registered in the 7–12 age group (11-year-old boy, a school dropout, from a disorganized family with a history of intoxication with psychodysleptic substances).

Cleaners and detergents were identified in 60 cases (7.52% of all poisonings), being an accidental ingestion in most cases (56 cases), predominantly in rural areas (36 cases), in males (33 patients), and in the 1–3-year-old age group.

Corrosive substances, often sodium hypochlorite, were involved in 67 cases (8.4% of poisonings), being an accidental type of poisoning (64 patients), predominantly occurring in rural areas (43 cases), in males (42 patients) in the 1–3-year-old age group (36 cases), followed by the 3–7-year-old age group. In the 12–18-year-old age group, corrosive substances (sodium hypochlorite, caustic soda, household disinfectants) were used for suicide, predominantly by females.

Nitrite poisoning is a severe type of poisoning with often harmful consequences, resulting from the use of water contaminated with nitrites for the preparation of powdered milk or food. In the studied group, 14 cases were registered in the 0–1-year-old age group, all coming from rural areas. Two patients had cardiopulmonary arrest, being resuscitated at home by the ambulance crew, with methemoglobin values in these patients being over 85% of total hemoglobin. Of these 14 poisonings, 5 cases were severe, 6 were moderate and 3 were mild. Rapid recognition of intoxication and prompt use of the antidote have led to favorable case evolutions.

Exposure to various medicines used by family members or to existing medicines at home was recorded in 75 patients (9.41% of cases). This was often voluntary exposure in patients in the 12–18 age group (27 patients), followed by accidental intoxication in the age group 1–3 years and 3–7 years (21 patients each). The most common medications included mucolytics, vasodilators, retardants, sartans, and oral antidiabetics. Neuro-psychiatric medication was identified in 68 patients, with 60.2% being voluntary intoxications, the patients being predominantly females from rural areas.

## 4. Discussion

Many adults consider childhood a time which, although irresponsible, is also carefree, neglecting the primary symptoms of anxiety or depression. Lack of adequate support to alleviate the suffering associated with these conditions and the failure of those around them to understand and make sense of the reality they are going through, and provide adequate support for the child in such a situation, has eventually led to substance abuse or voluntary poisonings with suicidal intent.

The prognoses and consequences for poisoned children depend on various factors, including the type of toxic substance involved, amount of the substance ingested, the age and overall health of the child, and the speed at which treatment is received. In some cases, children may experience only minor symptoms that resolve quickly with appropriate treatment. In other cases, poisoning can lead to serious health consequences, including organ damage, long-term disability, or even death. It is important to seek medical attention as soon as possible if a child is suspected of having been poisoned, as prompt treatment can greatly improve the outcome.

It is well known that the pandemic triggered by the SARS-CoV-2 virus had a strong impact on the number of cases that have addressed ED. Thus, it was found that the incidence of pediatric patients intoxicated with various substances was lower in 2020 compared to 2019 (446 versus 351), but higher in percentage compared to the annual number of presentations (1.19% versus 1.92%). Regarding the seasonal variations, we identified a lower incidence of cases in spring of 2020 compared to 2019 (20.86% versus 27.96%), which could be explained by the state of emergency imposed by the SARS-CoV-2 pandemic that limited travel and led to closer surveillance of pediatric patients. These percentages were similar to those identified in other studies regarding the number of admissions for poisoning in pediatric ED during the SARS-CoV-2 pandemic [1].

The frequency of intoxication varies with age and is the result of complex interactions between the following factors: toxic substances, the child’s specificity, and the child’s family environment. The adolescence is marked by transitions such as physical, emotional and social changes. The curiosity of young people, their desire to experience new things and the influence of their entourage can lead them to make choices that could endanger their lives. Additionally, suicidal tendencies in adolescents in a psycho-emotional and social context complete the palette of the adolescence period. These aspects are supported by the increased incidence of intoxications, with the 12–18-year-old age group accounting for 49.44% of this category. Those aged between 1–3 years account for 23.34% of intoxications, an aspect that supports the stages of normal neuropsychic development—that is, children taking their first steps and undertaking discovery of things around their environment. According to the published literature, the highest incidence of poisoning occurs in the first 3 years of life, especially in children aged 1–2 years [2]. The young child (1–4 years) is susceptible to accidental intoxication with drugs or toxic substances that are improperly stored or easily reachable due to the lack of attention by family members. Voluntary poisoning occurs after the age of 12, being characterized by a higher severity and with an increased risk of death [3]. This study found there was an increased rate of female poisoning for the 12–18-year-old age group. This can be explained by the increased tendency to internalize emotions and increase in the incidence of behavioral problems, along with the prevalence of suicidal tendencies, often aimed at raising awareness of the family, using non-violent methods and substances with low toxicity.

Some toxic substances can cause specific health consequences in children. For example, lead poisoning can cause developmental delays, behavioral problems, and neurological issues. Carbon monoxide poisoning can cause brain damage and long-term cognitive problems. Opioid poisoning can cause respiratory depression, which can be life-threatening. Ingestion of certain types of mushrooms can cause severe liver damage. It is important to identify the specific toxic substance involved in order to provide the most appropriate treatment and assess the potential consequences

Children exposed to toxic substances may exhibit various clinical symptoms (such as pulmonary, cardiovascular, neurological, or gastrointestinal issues) that can help us identify the toxic substance involved.

The main factor that determines a respiratory diagnosis is the change in respiratory rate. In children, hypoxia is more common than arrhythmias in poisonings, while in adults it is the opposite. Certain substances can affect the respiratory rate in different ways: salicylates can stimulate the respiratory center and cause hyperventilation and respiratory alkalosis; opioids suppress the respiratory center; venoms and irritant gases can cause glottic edema and bronchospasm; strychnine causes tetanic contraction of the respiratory muscles; atropine and barbiturates can lead to paralysis of the bulbar centers; and nitrates, nitrites, and carbon monoxide can prevent hemoglobin from binding oxygen.

The cardiovascular system can be affected in a number of ways by toxic substances. These effects include hypertension (caused by imidazoles, amphetamines, cocaine, nicotine, ergotamine, or monoamine oxidase inhibitors), arterial hypotension (caused by beta-adrenergic antagonists, calcium blocker antagonists, digoxin, diuretics, methylxanthines, salicylates, organophosphates, arsenic, clonidine, nitrates, or tricyclic antidepressants), bradycardia (caused by clonidine, methyldopa, beta-adrenergic antagonists, calcium channel antagonists, cholinergic agents, digoxin, or opioids), and tachycardia (caused by cocaine, amphetamines, salicylates, carbon monoxide, theophylline, or anticholinergics).

Ingesting a toxic substance in sufficient dosage can impair consciousness or even cause coma. Therefore, the neurological examination is crucial in determining the diagnosis. Symptoms that may be identified include altered mental status and convulsions (caused by isoniazid, theophylline, salicylates, digoxin, tricyclic antidepressants, organophosphates, mushrooms, or snake bites). Abnormal movements may also be present, such as choreiform movements (caused by anticholinergic agents, anticonvulsants, carbon monoxide, or lithium), tremors (caused by caffeine, theophylline, amphetamines, phenytoin, valproic acid, carbamazepine, or cocaine), or dyskinesia or dystonia (caused by metoclopramide or antipsychotics).

Clinical gastroenterological manifestations in child poisonings can include nausea, vomiting, abdominal pain, diarrhea, and constipation. Other symptoms may include loss of appetite, weight loss, abdominal swelling, and jaundice. Some toxic substances can also cause gastrointestinal bleeding, which can lead to black or tarry stools, or rectal bleeding. In severe cases, children may experience dehydration or electrolyte imbalances as a result of the poisoning. Some toxic substances can also cause damage to the liver or other internal organs, which may manifest as additional symptoms.

Regarding the origin environment of patients, most come from rural areas (65.9%, 525 out of 797 patients). Precarious socio-economic conditions, lack of education, and disorganized family structures due to the departure of one or both parents to work abroad are often the causes, as well as the variety of toxins to which rural patients may be exposed compared to those in urban areas. These percentage variations are influenced by the patient’s environment of origin (rural environments often resulting in limited accessibility to transport), but also by the fact that some of the patients were initially evaluated in other health units.

Of all intoxicated patients, 13.04% were considered special situations (disorganized families, family alcoholism, school dropout, anxiety-depressive disorders, early pregnancy). These situations can have a negative effect on a young person at an individual psychological level, notably in altering their self-image, with a consequent loss of confidence in one’s own possibilities and abilities. In addition, in social terms, permanent school failure or belonging to a disorganized family can lead to stigmatization, labeling, and/or social marginalization, resulting in a high level of deviant and criminal behavior. To prevent these situations, a complex approach is needed, including integrated policies aimed at sex education, greater community awareness of the specific needs of adolescents, and the provision of services tailored to the psychological and emotional profile of teenage women [4].

Our study found that 561 out of the 797 pediatric poisonings studied occurred at home, similar to other studies. This confirms the high availability of potentially toxic substances in the family environment (for example, chronic treatments for family members, alcohol, cleaning agents), inadequate storage of toxic products, and the attractive appearance of containers or their contents, often associated with moments of negligence of the child [5].

Accidental intoxications are the primary cause (50.19%), being typical of pediatric age. Their percentage decreases according to the emotional and cognitive development of the patients, while the incidence of voluntary intoxications, which are seen as a possible escape route for the adolescent’s problems, begins to increase. This occurs in conjunction with increasing awareness of the concept of death. In the case of voluntary intoxications, they were highlighted mainly in the 12–18 age group, with numerous causes. Most of the time, however, they represented a cumulation of notable events, with an important emotional impact in the adolescents’ life. Problems and conflicts in the family or at school, romantic disappointments, and lack of self-acceptance can lead to indiscriminate behavior and suicide attempts [4]. In all voluntary intoxications, after stabilization of the patient, a careful psychological evaluation and, where appropriate, psychiatric consultation is required.

The most common route of administration was oral, being an easily-accessible method, and with most toxins available in solid or liquid form, they are easily procured and ingested, especially for adolescents (alcohol consumption or use of substances with increased toxicity for suicidal use). Young children are susceptible to ingestion of toxic substances, especially liquids, with an attractive presentation, the perception at this age being mainly visual and gustatory. The inhalation route predominated in the 12–18-year-old age group, as they made up some 60% of the total number of patients in this category (recreational drugs being the ones that dominate the area of inhaled toxins), followed by the 7–12-year-old age group, which accounted for 16.84%, featuring a higher incidence for male patients.

Young children are susceptible to intoxication with various potentially toxic solutions existing in the household, or with medication from family members. They are attracted by the colorful and attractive packaging of many household products. Thus, toxic substances such as cleaning substances (detergents), personal care substances (body cream, nail polish), insecticides (often stored in inappropriate containers), various medicines (statins, contraceptives, hepatoprotectants, vasodilators, neuropsychiatric medication, antibiotics, antipyretics) can frequently be discovered by children. There are differences between the percentage of intoxications by cleaning substances identified in this study (7.52%) compared to the results of other European studies (49%), but results were similar regarding exposure to pharmaceuticals [6]. Exposure to cleaners and detergents was facilitated by a lack of family supervision, the commercially-attractive appearance of the packaging of these substances (brightly-colored detergent pads), and the curiosity of young children. In elderly patients, suicidal use of cleaning substances has been identified in females from rural areas. The prognoses and consequences for children poisoned by cleaners and detergents depends on various factors, including the specific cleaner or detergent involved, the amount ingested, the age and overall health of the child, and the speed at which treatment is received. In general, cleaners and detergents can cause a wide range of symptoms in children, including nausea, vomiting, abdominal pain, and diarrhea. Some cleaners and detergents can also cause irritation to the skin, eyes, and respiratory system. In severe cases, children may experience difficulty breathing, coughing, or chemical burns. Treatment may include medications to control vomiting, activated charcoal to absorb the toxic substance, and supportive care to manage symptoms. In severe cases, hospitalization may be necessary. The long-term consequences of cleaner and detergent poisoning may include organ damage, long-term disability, or even death. It is important to take preventive measures to avoid accidental poisonings by cleaners and detergents. This includes keeping all cleaning products out of reach of children, using child-resistant packaging whenever possible, and properly labeling and storing cleaners and detergents.

The analysis of cases of intoxication with ethyl alcohol from a biological point of view (alcoholemia) showed there is no definite correlation between blood alcohol levels and the degree of intoxication (clinical condition of patients, including neurological status), possibly due to tolerance phenomena. It is important to note that the clinical symptoms of alcohol intoxication differ between children, adolescents, and adults. Until laboratory results are available, the diagnosis will be based on clinical observations and will guide the treatment plan. In young children, ethanol ingestion is often associated with hypoglycemic status, respiratory depression, and sedative effects. In adolescents, ethanol consumption initially causes a state of euphoria, followed by respiratory depression. Hypoglycemia is less common in adolescents than in young children due to lower liver glycogen stores. In the study group, patients intoxicated with ethyl alcohol were often victims of a traumatic pathology, either due to the neurological impairment secondary to intoxication, aggression, or road traffic accidents. The percentage of cases with alcohol intoxications as well as the predominance of the male gender in adolescents was similar to that found in other studies [7,8,9]. Alarming is the increasing number of ethanol-intoxicated patients, their young age, the high level of blood ethanol concentrations, and the severe symptoms of these patients. This is the reason why early and intensive prevention strategies are required.

Acute pesticide poisoning in children is rare but potentially serious. Some clinical patterns (toxidromes) are suggestive of the drug class: cholinergic crisis for organophosphate or carbamate insecticides; neurological syndrome for rodenticides; digestive and respiratory syndrome for herbicides. Treatment is symptomatic and only a few patients are treated with an antidote: atropine and pralidoxime for organophosphate insecticides, or vitamin K for anticoagulant rodenticides [10]. The long-term consequences of pesticide poisoning may include organ damage, long-term disability, or even death. Thus, it is important to take preventive measures to avoid accidental poisonings by pesticides.

As the number of states legalizing psychoactive substances for medical and/or recreational use continues to grow, there are an increasing number of children exposed to products containing marijuana in homes and communities. Increased exposure leads to a greater probability of accidental ingestion and toxicity. Because ingestion of this type of drugs can cause a dangerous and potentially life-threatening toxicity for children, pediatric health care providers need an increased awareness of the danger [11]. The difficulties in diagnosing intoxication with psychoactive substances mean this type of intoxication can be underestimated [12]. Currently, in the ED, the diagnosis for this type of intoxication is based on anamnesis and the clinical picture, the identification of the incriminated substance being impossible (and even more so for the new synthetic drugs). Brain injury in case of neuro-psychiatric medication was mild in 76.46% of cases, proof that the toxic doses were not high, with the emotional aspect of intoxication instead being paramount.

In adolescent patients, poisoning is often suicidal, so the toxic substances to which they have been exposed are neuropsychiatric medications, multidrug combinations, drugs associated with alcohol, or insecticides. Suicidal ideation indicates a high level of emotional lability and is a worrying issue with a negative impact on the family, the individual, and also society, requiring specialized psychiatric consultation. Suicidal ideation is often a result of underlying mental health issues, such as depression, anxiety, or trauma. Alcohol and recreational drugs are common toxins in adolescent patients who have received medical care. Easy access to these substances, the degree of education, family example, the desire to ‘impress the entourage’, or the need to integrate into a certain social group, are common reasons in this type of intoxication [13].

Generally, substances found in pediatric intoxications have a low degree of toxicity, but there are also situations where the degree of toxicity is high, requiring immediate and appropriate medical intervention to prevent severe complications or even death. To further reduce the number of deaths and disabilities in the industrialized world and to begin to have an effect in the developing world, much more work is required to both identify and implement prevention strategies to reduce the number of cases of pediatric poisoning [14].

Preventive measures are crucial in preventing acute poisoning in children. Acute poisoning is the ingestion of a toxic substance in a single exposure or over a short period of time, and it can be life-threatening if not treated promptly. Children are particularly vulnerable to acute poisoning because they are more likely to explore their environment by putting things in their mouths. Therefore, it is important to take steps to prevent accidental poisonings in children. One of the most effective preventive measures is to keep all toxic substances out of reach of children. This includes medications, cleaning products, pesticides, and any other potentially harmful products. It is also important to use child-resistant packaging whenever possible, as these designs make it difficult for children to open the container.

It is important to educate children on the dangers of ingesting unknown substances and to teach them to ask an adult before consuming anything. Another preventive measure is to keep the National Capital Poison Center’s poison prevention tips in mind: “Keep it up high and out of sight, keep it locked up tight, and keep it out of reach when you’re not around.” This can help to prevent accidental poisonings in children.

It is also important to store medications in their original packaging and to label them properly. Medications should be disposed of properly, according to the guidelines provided by the manufacturer or the local waste management agency. Keeping a list of all medications at home, including the name, dosage, and purpose of each medication, can be helpful in case of accidental poisoning. Finally, in the case of children with special needs, such as a developmental delay or cognitive impairment, it is especially important to be vigilant in keeping toxic substances out of reach.

Pediatric toxicology presents unique challenges due to the increased incidence of poisonings in the 1–3-year age range. In these cases, it can be difficult to accurately diagnose the poisoning, as the child may not be able to accurately communicate what they ingested, how much they ingested, and when they ingested it. It is important to carefully consider the maximum potential dose that the child may have taken, as there may be uncertainty about the actual dose. Additionally, if there is more than one child present, it may be unclear who ingested the substance, so it is important to assume the worst-case scenario and treat the situation as if one child consumed as much as possible. To properly diagnose and treat pediatric toxicology cases, it is helpful to gather specific information about the timing and symptoms of the poisoning, as well as to use online resources such as the National Poisons Information Service to identify the substance involved.

Despite the economic challenges faced by the region where the study was conducted, we found that the rural environment had a higher incidence of intoxications in children. One might assume that access to information and resources would be limited in rural areas, but our study shows that patients from rural areas had a higher incidence of these issues compared to those from urban areas. This may be due to the rural environment and its associated challenges, such as the distance to hospitals, lack of transportation, and limited availability of ambulances, which can lead to delays in seeking medical attention. Additionally, lower educational levels may also contribute to the increased time interval between the moment of ingestion and presentation at the hospital.

Our study found that the time spent in the pediatric emergency department was shorter than in the adult emergency department, and the hospital admission criteria were wider for certain conditions. Additionally, we discovered there are substances that are not classified as drugs but can be potentially lethal if ingested.

According to Eurostat, Romania ranks second in the European Union (EU) for episodes of excessive alcohol consumption at least once a month, significantly exceeding the EU average.

The rate of global mortality due to poisoning in children is increasing alarmingly [2,5,13]. All medications, including vitamins, can be harmful if taken in large doses, and it is important for parents to be aware of the symptoms of poisoning, stay calm, and take appropriate action.

The general approach to managing acute poisoning should follow the RRSIDEAD principles: resuscitation, risk assessment, supportive care, investigations, decontamination, enhanced elimination, antidotes, and disposition. These principles provide a framework for effectively responding to and treating cases of acute poisoning [15].

## 5. Conclusions

Acute intoxications in children represent a small percentage of the admissions to the ED of the St Mary Children’s Hospital, Iași (1.43% of total submissions). In the studied group, acute intoxications were frequently identified in the 12–18-year-old age group (49.44%), followed by the 1–3-year-old age group (23.34%), especially in male patients (51.7%), from rural areas (65.9%).

The timing of arrival in the ED is essential, with the patient’s prognosis practically depending on this time interval. In the study group, the average time from exposure to the first medical evaluation was 3.53 h, a small percentage coming in the first hour after the incident.

Regarding the type of intoxication, the accidental one slightly predominates (50.2% of the total) compared to the voluntary ones (47.4%), with both types of intoxication having a higher incidence in rural patients (64.7%).

Some 70.4% of intoxications occurred at the patient’s home, 69.5% of which were accidental. Most poisonings in another location (95.6%) were voluntary. An increased percentage of voluntary intoxications was registered in the 12–18-year-old group, and the accidental ones predominated in the 1–3-year-old group. Most cases of intoxication, both voluntary and accidental, were oral (87.1%). Inhalation poisoning, although lower in percentage (11.9%), is statistically important through its social implications (exposure to illicit substances), but also in educational terms (carbon monoxide poisoning).

The toxins to which the patients from the studied group were exposed are extremely varied, being an image of the patient’s social and economic life. Our study also found that alcohol consumption, either alone or in combination with other toxins such as antipsychotics and anticonvulsants, is the main criminalized substance among children, especially in 12–18-year-old patients. The factors that influence heavy alcohol consumption include the environment in which children live and sellers of alcoholic beverages who do not follow the legal age limit.

Accidental exposure to cleaning substances and corrosive substances is common in young patients, but the same toxicants are also used for suicidal purposes by adolescents. Although the percentage of the exposure to these corrosive substances is low (8.4%), the clinical implications are severe, with significant complications both immediate and distant, and frequently require hospitalization. Illicit drug intoxication is often underestimated, either due to the difficulties of toxicological identification in the ED, or by reduced accessibility to the medical services.

Identifying and documenting epidemiological aspects and other variables are important for establishing preventive measures, but also for therapeutic conduct. Adequate risk stratification and preventive measures involving closer supervision of minors or cognitive behavioral programs can prevent voluntary intoxication, especially in at-risk categories.

## Figures and Tables

**Table 1:** Incidence of poisoning by age groups in the studied group.

Age	Frequency	Percent	Valid Percentage	Cumulative Percentage
0–1 years	44	5.5	5.5	5.5
1–3 years	186	23.3	23.3	28.9
3–7 years	107	13.4	13.4	42.3
7–12 years	66	8.3	8.3	50.6
12–18 years	394	49.4	49.4	100.0
Total	797	100.0	100.0	

**Table 2:** Correlation age-residence-gender in the study group.

		Age	Gender	Residence
Age	Pearson Correlation	1	−0.124 **	−0.030
	Sig. (2-tailed)		0.000	0.395
	N	797	797	797
Gender	Pearson Correlation	−0.124 **	1	0.104 **
	Sig. (2-tailed)	0.000		0.003
	N	797	797	797
Residence	Pearson Correlation	−0.030	0.104 **	1
	Sig. (2-tailed)	0.395	0.003	
	N	797	797	797

**. Correlation is significant at the 0.01 level (2-tailed).

**Table 3:** Type of poisoning.

	Frequency	Percentage	Valid Percentage	Cumulative Percentage
Accidental	400	50.2	50.2	50.2
Voluntary	378	47.4	47.4	97.6
Iatrogenic	18	2.3	2.3	99.9
Unknown	1	0.1	0.1	100.0
Total	797	100.0	100.0	

## Data Availability

The raw data supporting the conclusions of this article are freely available from the authors upon request.
